# Salidroside Ameliorates Renal Interstitial Fibrosis by Inhibiting the TLR4/NF-κB and MAPK Signaling Pathways

**DOI:** 10.3390/ijms20051103

**Published:** 2019-03-04

**Authors:** Rui Li, Yujuan Guo, Yiming Zhang, Xue Zhang, Lingpeng Zhu, Tianhua Yan

**Affiliations:** 1 Department of Physiology and Pharmacology, School of basic medicine and clinical pharmacy, China Pharmaceutical University, 24 Tongjiaxiang, Nanjing 210009, China; lr082261@163.com (R.L.); yujuanguo@vip.163.com (Y.G.); 15298360632@163.com (Y.Z.); abc18851107671@163.com (X.Z.); 2 Department of Biochemistry, School of Life Science and Technology, China Pharmaceutical University, 24 Tongjiaxiang, Nanjing 210009, China

**Keywords:** salidroside, renal interstitial fibrosis, epithelial-mesenchymal transition, TLR4, inflammation

## Abstract

Salidroside (Sal) is an active ingredient that is isolated from *Rhodiola rosea*, which has been reported to have anti-inflammatory activities and a renal protective effect. However, the role of Sal on renal fibrosis has not yet been elucidated. Here, the purpose of the current study is to test the protective effects of Sal against renal interstitial fibrosis (RIF), and to explore the underlying mechanisms using both in vivo and in vitro models. In this study, we establish the unilateral ureteric obstruction (UUO) or folic acid (FA)-induced mice renal interstitial fibrosis in vivo and the transforming growth factor (TGF)-β1-stimulated human proximal tubular epithelial cell (HK-2) model in vitro. The levels of kidney functional parameters and inflammatory cytokines in serum are examined. The degree of renal damage and fibrosis is determined by histological assessment. Immunohistochemistry and western blotting are used to determine the mechanisms of Sal against RIF. Our results show that treatment with Sal can ameliorate tubular injury and deposition of the extracellular matrix (ECM) components (including collagen Ш and collagen I). Furthermore, Sal administration significantly suppresses epithelial-mesenchymal transition (EMT), as evidenced by a decreased expression of α-SMA, vimentin, TGF-β1, snail, slug, and a largely restored expression of E-cadherin. Additionally, Sal also reduces the levels of serum biochemical markers (serum creatinine, Scr; blood urea nitrogen, BUN; and uric acid, UA) and decreases the release of inflammatory cytokines (IL-1β, IL-6, TNF-α). Further study revealed that the effect of Sal on renal interstitial fibrosis is associated with the lower expression of TLR4, p-IκBα, p-NF-κB and mitogen-activated protein kinases (MAPK), both in vivo and in vitro. In conclusion, Sal treatment improves kidney function, ameliorates the deposition of the ECM components and relieves the protein levels of EMT markers in mouse kidneys and HK-2 cells. Furthermore, Sal treatment significantly decreases the release of inflammatory cytokines and inhibits the TLR4/NF-κB and MAPK signaling pathways. Collectively, these results suggest that the administration of Sal could be a novel therapeutic strategy in treating renal fibrosis.

## 1. Introduction

Chronic kidney disease (CKD) remains a leading public health issue, being the third highest cause of premature mortality (82%), behind AIDS and diabetes mellitus [1,2]. It has been predicted that about 160 million individuals will be affected by CKD by 2020 [3]. Despite the seriousness of this problem, there are not enough therapeutic options for CKD in the clinical setting. Therefore, there is a need for people to find more effective therapeutics to treat CKD and reduce healthcare expenditure. Meanwhile, understanding the mechanisms behind renal interstitial fibrosis is essential for developing therapies to prevent or reverse the progression of CKD.

Renal interstitial fibrosis (RIF) is the final common outcome of CKD and ultimately end-stage renal failure [4]. The histopathology of renal interstitial fibrosis features deposition of the extracellular matrix (ECM) components, loss of tubular cells, accumulation of fibroblasts, and rarefaction of the peritubular microvasculature [5]. The ECM components include collagen I and collagen Ш. Epithelial-mesenchymal transition (EMT) is the most important cause of renal interstitial fibrosis and is characterized by renal tubular epithelial cells that acquire mesenchymal phenotypes and myofibroblast functions [6]. The transition of EMT induces kidney epithelial cells to decrease the expression of adherens junction proteins such as E-cadherin, and strongly induces the expression of fibroblast markers, including vimentin and α-smooth muscle actin (α-SMA) [7]. TGF-β1, snail, and slug are important profibrotic mediators for EMT [8]. Renal fibrosis is almost always evolved by the infiltration of inflammatory cells, including increased inflammatory cytokines (TNF-α, IL-6, and IL-1β). Although inflammation is an integral part of the host defense mechanism response to injury, nonresolving inflammation is a major driving force in the development of fibrotic disease [9].

Toll-like receptor 4 (TLR4) is an important mediator of inflammation in the kidney. It has been reported that TLR4 mediates both pro-inflammatory and pro-fibrotic pathways in renal fibrosis [10]. Lipopolysaccharide (LPS) is a primary ligand for the TLR4 receptor and studies have shown that LPS induces the aggregation of TLR4 on HK-2 cells, promoting an inflammatory response [11]. Nuclear factor-kappa B (NF-κB) is also known to be important in the expression of pro-inflammatory genes, and treatment with the NF-κB inhibitor pyrrolidine dithiocarbamate (PDTC) shows attenuated renal injury and inflammation in animal models of CKD [12]. These findings reveal that NF-κB plays a pivotal role in the progression of chronic renal inflammation. The mitogen-activated protein kinases (MAPK) signaling pathway, including JNK, Erk, and Ρ38, is involved in the production of pro-inflammatory and pro-fibrotic mediators. The MAPK pathway is activated by multiple stimulations, including IL-1β and TNF-α, which cause the translocation of cytoplasmic nuclear factor-κB (NF-κB) from nucleus to active NF-κB. In addition, MAPK activation has been reported to be involved in the secretion of TGF-β1 and ECM proteins [13].

**Figure 1:**
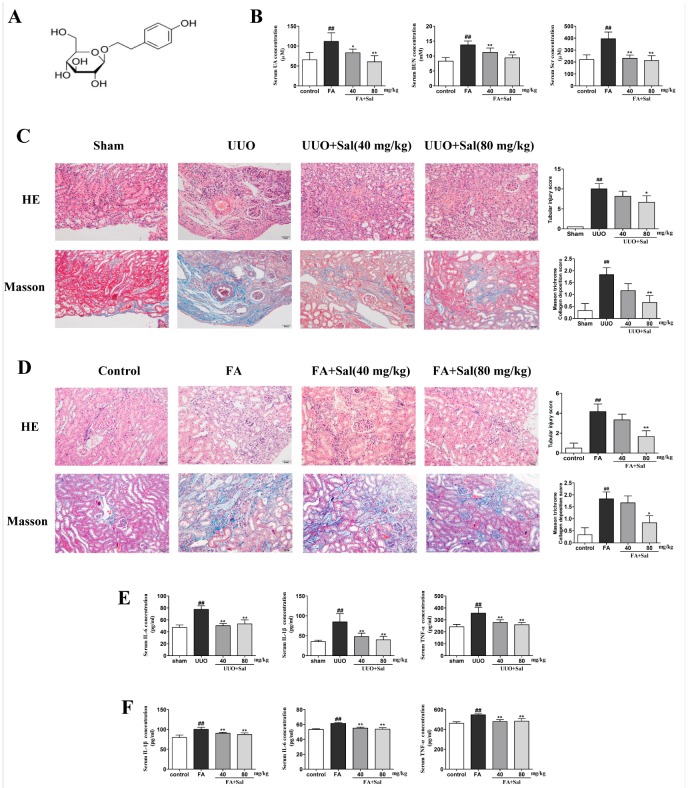
Sal alleviated renal function parameters, histopathology and inflammation in renal fibrotic mice. (**A**) The structure of salidroside (Sal). (**B**) Sal decreases the serum levels of serum creatinine (Scr), blood urea nitrogen (BUN) and uric acid (UA) in FA mice (*n* = 8). (**C**) Representative images of hematoxylin and eosin (H&E) and Masson staining of kidney tissue in unilateral ureteric obstruction (UUO) mice (*n* = 3). Original magnification: ×200. (**D**) Representative images of H&E and Masson staining of kidney tissue in folic acid (FA) mice (*n* = 3). The score of the lesion was determined to be either 0.5 points (slight or very little), 1 point (mild or small), 2 points (moderate or more), 3 points (severe or more quantity), or four points (extremely severe or a lot). When there was no obvious lesion, the score was 0 points. Original magnification: ×200. (**E**) The serum levels of TNF-α, IL-1β, and IL-6 in UUO mice were determined using enzyme-linked immunosorbent assay (ELISA) kits (*n* = 6). (**F**) The serum levels of TNF-α, IL-1β, and IL-6 in FA mice were determined using ELISA kits (*n* = 6). All data are represented as mean ± SD. ^#^ P < 0.05, ^##^ P < 0.01 vs. Sham group, * P < 0.05, ** P < 0.01 vs. UUO group; ^#^ P < 0.05, ^##^ P < 0.01 vs. control group, * P < 0.05, ** P < 0.01 vs. FA group.

Salidroside (Sal, p-hydroxyphenethyl-bD-glucoside, C_14_H_20_O_7_, structure shown in Figure 1A), one of the bioactive compounds extracted from *Rhodiola rosea* L., has many pharmacological effects, such as anti-cancer [14], anti-depressive [15], anti-inflammatory [16], anti-oxidant [17], anti-ulcer [18] and cardioprotective [19]. However, there have been no reports on Sal in the context of renal interstitial fibrosis. Therefore, this study aims to provide insight into the therapeutic effects of Sal in the context of renal interstitial fibrosis and attempts to explore the molecular mechanisms.

## 2. Results

### 2.1. Sal Alleviated Renal Function Parameters, Histopathology and Inflammation in Renal Fibrotic Mice

The serum levels of serum creatinine (Scr), blood urea nitrogen (BUN), and uric acid (UA) are the classical indicators of renal function. The serum concentrations of UA, Scr, and BUN were significantly increased in the folic acid (FA) mice. Sal reduced the serum levels of UA, Scr, and BUN in renal fibrotic mice (Figure 1B). Hematoxylin and eosin (H&E) staining revealed renal structural damage, inflammatory cell infiltration and extracellular matrix deposition in the unilateral ureteric obstruction (UUO) and FA mice, which was significantly restored by Sal (Figure 1C,D). Additionally, Masson’s staining showed a large number of collagen fiber streaks, staining blue with prominent collagen fiber hypertrophy in the UUO and FA mice. However, fewer collagen fiber streaks (blue stained area) were observed when the mice were treated with Sal (Figure 1C,D). These results indicated that Sal ameliorated renal damage and fibrosis in the fibrotic mice.

In order to investigate the inflammatory alterations in RIF, we detected the levels of inflammatory factors IL-1β, IL-6 and TNF-α in the UUO and FA mice. As expected, all factors notably increased in the serum of UUO and FA mice, indicating that inflammation plays an important role in the development of renal fibrosis. Consistently, Sal remarkably decreased the levels of IL-1β, TNF-α, and IL-6 in the serum of the UUO and FA mice (Figure 1E,F), indicating that Sal could inhibit the inflammatory response in RIF.

### 2.2. Sal Inhibited ECM Deposition and EMT in Renal Fibrotic Mice

**Figure 2:**
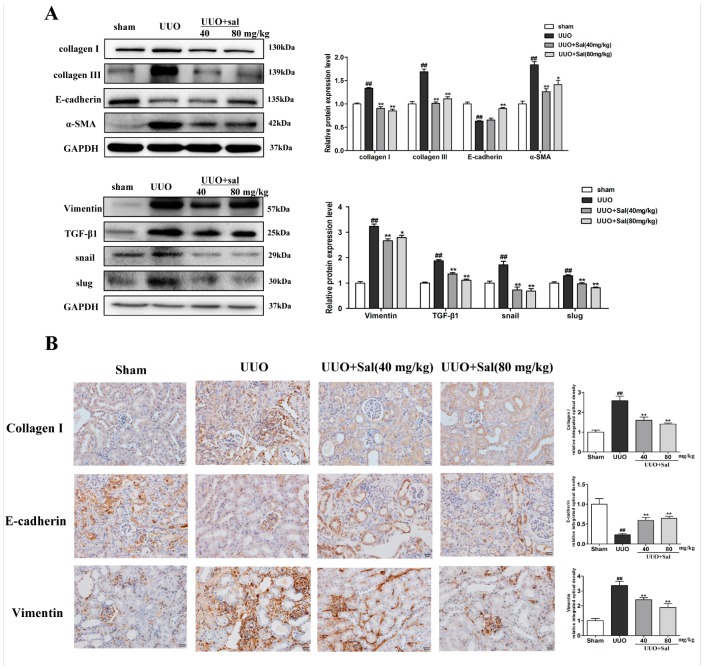
Sal reduced the accumulation of extracellular matrix components and blocked EMT in UUO mice. (**A**) Western blotting was performed to determine the protein expression of collagen I, collagen Ш, α-SMA, vimentin, E-cadherin, TGF-β1, snail, and slug in the kidney tissue of UUO mice (*n* = 3). (**B**) Immunochemical staining of collagen I, E-cadherin, and vimentin in the kidney tissue of UUO mice (*n* = 3). Original magnification: ×400. All data are represented as mean ± SD. ^#^ P < 0.05, ^##^ P < 0.01 vs. Sham group, * P < 0.05, ** P < 0.01 vs. UUO group.

To evaluate whether Sal could affect the levels of ECM and fibrosis markers, we performed western blotting and immunohistochemistry analysis on the kidney tissue. The primary feature of renal interstitial fibrosis is the accumulation of ECM components. Collagen I and collagen Ш are the main components of ECM. The levels of collagen I and collagen Ш were notably enhanced in the UUO mice, whereas these alternations were significantly inhibited by Sal (Figure 2A). Myofibroblasts are considered to be a primary renal interstitial cell, responsible for production of ECM during fibrosis. Furthermore, EMT activation has been associated with myofibroblast production. In the present study, Sal was found to downregulate α-SMA and vimentin protein levels, and upregulate E-cadherin protein levels in UUO mice (Figure 2A). TGF-β1, snail, and slug are key regulators of the EMT program and RIF [20], whereas Sal attenuates the UUO-induced upregulation of TGF-β1, snail, and slug in UUO mice.

In the immunohistochemistry analysis, the effect of Sal on the expressions of collagen I, vimentin, and E-cadherin in the kidney tissue was further examined (Figure 2B). Sal downregulated the collagen I and vimentin protein levels and upregulated the E-cadherin protein level in UUO mice.

**Figure 3:**
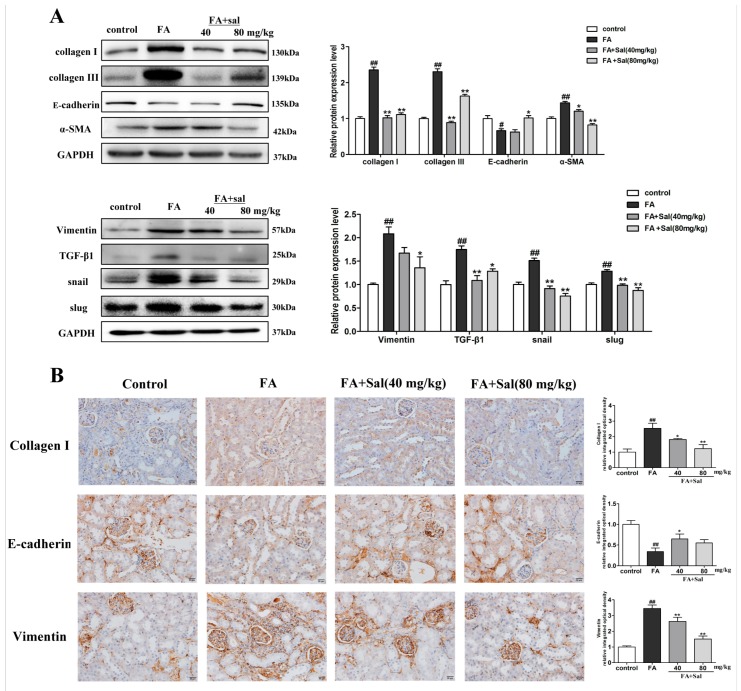
Sal reduced the accumulation of extracellular matrix (ECM) components and blocked EMT in FA mice. (**A**) Western blotting was performed to determine the protein expression levels of collagen I, collagen Ш, α-SMA, vimentin, E-cadherin, TGF-β1, snail, and slug in the kidney tissue of FA mice (*n* = 3). (**B**) Immunochemical staining of collagen I, E-cadherin and vimentin in the kidney tissue of FA mice (*n* = 3). Original magnification: ×200. All data are represented as mean ± SD. ^#^ P < 0.05, ^##^ P < 0.01 vs. control group, * P < 0.05, ** P < 0.01 vs. FA group.

The same effect of Sal was observed in the FA mice (Figure 3A,B). The results indicated that Sal could prevent the expression of ECM components and EMT markers.

### 2.3. Sal Suppresses Renal Inflammation via the TLR4/NF-κB and MAPK Signaling Pathways in Renal Fibrotic Mice

**Figure 4:**
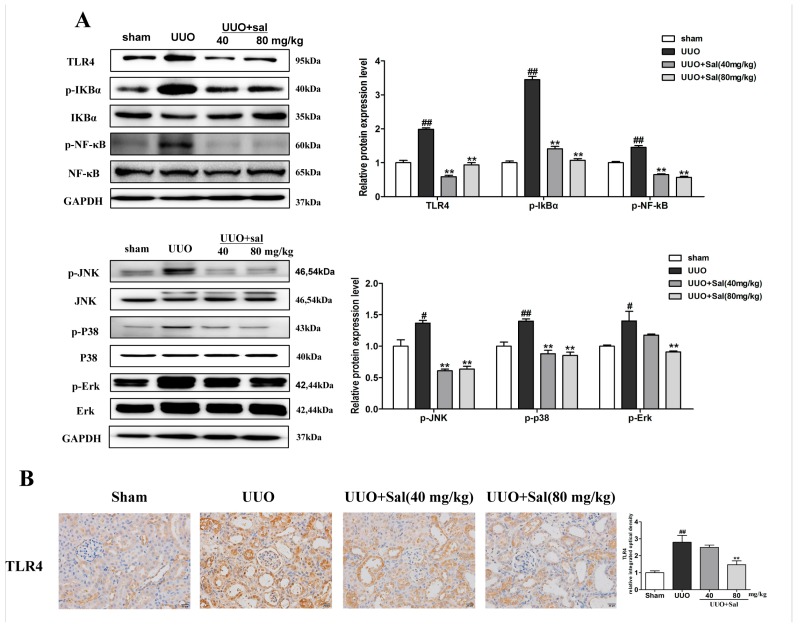
Sal suppresses renal inflammation via the TLR4/NF-κB and MAPK signaling pathways in UUO mice. (**A**) Western blot analysis of TLR4, p-IкBα, p-NF-κB, p-Ρ38, p-ERK, and p-JNK protein expression in the kidney tissue of UUO mice (*n* = 3). (**B**) Immunochemical staining of TLR4 in the kidney tissue of UUO mice (*n* = 3). Original magnification: ×200. All data are represented as mean ± SD. ^#^ P < 0.05, ^##^ P < 0.01 vs. Sham group, * P < 0.05, ** P < 0.01 vs. UUO group.

Inflammation contributes to the progression of renal interstitial fibrosis, as TLR4 induces the expression of inflammatory cytokines. As the promoter of a signaling pathway, TLR4 activates NF-кB and MAPK and also stimulates pro-inflammatory reactions [21]. In order to investigate the anti-inflammatory mechanism of Sal on renal interstitial fibrosis, western blot and immunohistochemistry experiments were conducted. In the western blotting, Sal significantly suppressed the expression of TLR4, p-IкBα, p-NF-κB, p-Ρ38, p-ERK, and p-JNK in UUO mice (Figure 4A). The immunohistochemistry experiment also was used in our study. The results of the experiment showed that Sal treatment significantly suppressed the expression of TLR4 in UUO mice (Figure 4B).

**Figure 5:**
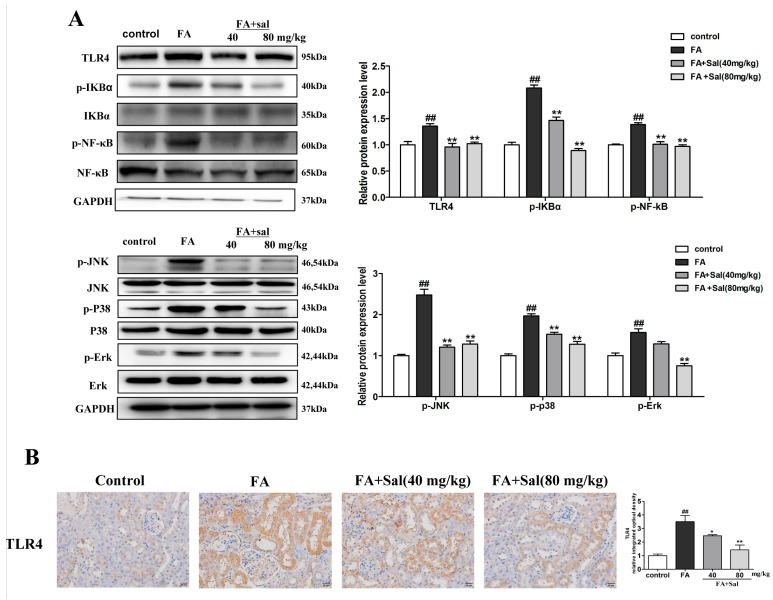
Sal suppresses renal inflammation via the TLR4/NF-κB and MAPK signaling pathways in FA mice. (**A**) Western blot analysis of TLR4, p-IкBα, p-NF-κB, p-Ρ38, p-ERK, and p-JNK protein expression in the kidney tissue of FA mice (*n* = 3). (**B**) Immunochemical staining of TLR4 in the kidney tissue of FA mice (*n* = 3). Original magnification: ×200. All data are represented as mean ± SD. ^#^ P < 0.05, ^##^ P < 0.01 vs. control group, * P < 0.05, ** P < 0.01 vs. FA group.

The same effect of Sal was observed in the FA mice (Figure 5A,B). In conclusion, the TLR4/NF-кB and MAPK signaling pathways might play a role in RIF, and Sal ameliorates RIF by inhibiting the TLR4/NF-кB and MAPK signaling pathways.

### 2.4. Sal Inhibited TGF-β1-Induced HK-2 Cells ECM Deposition, EMT and Inflammatory Response via the TLR4/NF-κB and MAPK Signaling Pathways

**Figure 6:**
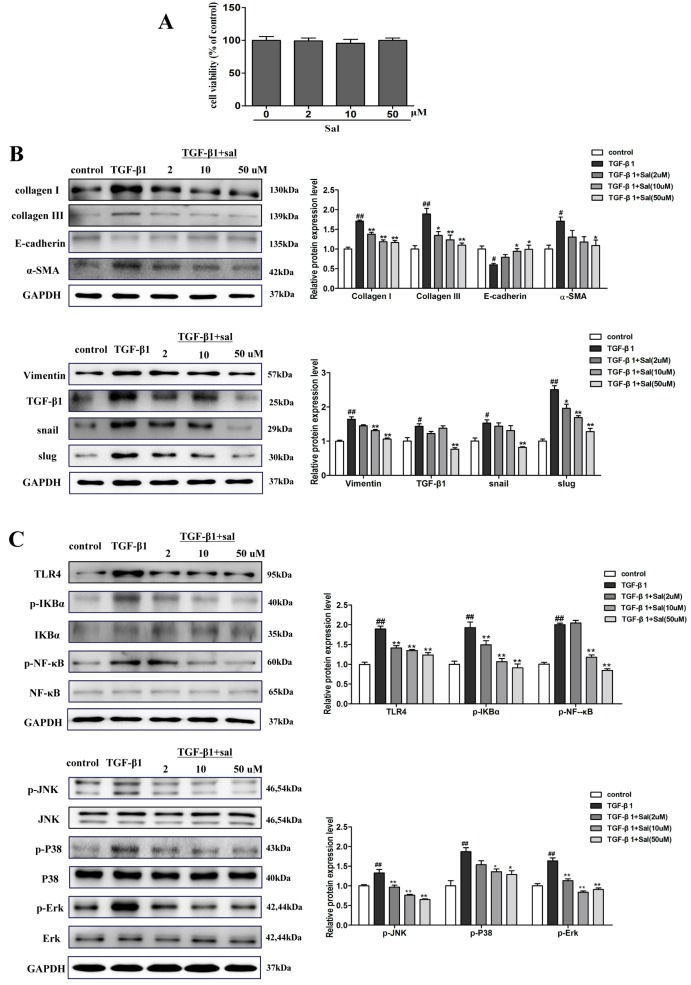
Sal inhibited TGF-β1-induced HK-2 cells ECM deposition, EMT and inflammatory response via the TLR4/NF-κB and MAPK signaling pathways. (**A**) Effect of different doses of salidroside on HK-2 cell viability. HK-2 cells (1 × 10^4^ cells/well) were exposed to a series concentration of salidroside (0, 2, 10, 50 μM) for 24 h. The viability of HK-2 cells was assessed by a cell counting kit-8 (CCK-8) assay. (**B**) Western blotting was performed to determine the protein expression levels of collagen I, collagen Ш, α-SMA, vimentin, E-cadherin, TGF-β1, snail, and slug in TGF-β1-induced HK-2 cells. The cell was incubated with different concentrations of Sal (2, 10, 50 μM), followed by stimulation with 5 ng/mL of TGF-β1 for 48 h. (**C**) Sal inhibited the expression of TLR4, p-IкBα, p-NF-κB, p-Ρ38, p-ERK, and p-JNK in TGF-β1-induced HK-2 cells. The cell was incubated with different concentrations of salidroside (2, 10, 50 μM), followed by stimulation with 5 ng/mL of TGF-β1 for 48 h. All data are expressed as mean ± SD, ^#^ P < 0.05, ^##^ P < 0.01 vs. control group, * P < 0.05, ** P < 0.01 vs. TGF-β1 group.

First, we assessed the cytotoxicity effect of salidroside on HK-2 cells. The results showed that Sal (2, 10, and 50 uM) treatment did not affect the viability of HK-2 cells (Figure 6A). We used a TGF-β1-induced HK-2 cell to detect the effect of Sal in vitro. TGF-β1 (5 ng/mL) obviously increased the protein expression level of collagen I, collagen Ш, α-SMA, vimentin, TGF-β1, snail, and slug in the model group, while E-cadherin was downregulated when compared with the control group. In agreement with the in vivo results, Sal (2, 10, 50 μM) significantly restored these alterations (Figure 6B). The results showed that Sal could prevent the expression of fibrosis markers and the ECM in TGF-β1-activated HK-2 cells.

In order to investigate the anti-EMT mechanism of Sal in TGF-β1-induced HK-2 cells, western blotting was conducted. The results demonstrated the upregulation of TLR4, p-IкBα, p-NF-κB, p-Ρ38, p-ERK, and p-JNK when compared with the control group, while the Sal treatment group effectively restored these alterations (Figure 6C). These results revealed that Sal suppresses the inflammatory response of TGF-β1-induced HK-2 cells by inhibiting the TLR4/NF-κB and MAPK signaling pathways.

### 2.5. Sal Suppressed the LPS-Induced Inflammatory Response by Inhibiting the TLR4/NF-κB and MAPK Signaling Pathways

**Figure 7:**
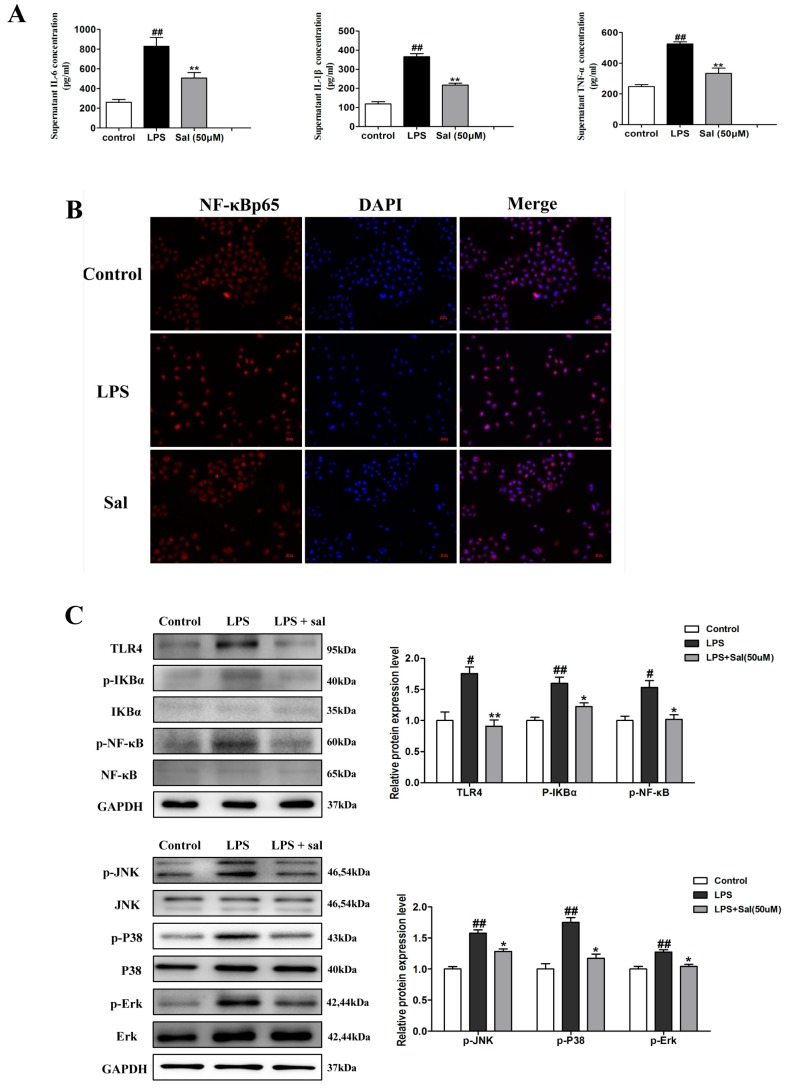
Sal suppressed lipopolysaccharide (LPS)-induced inflammatory response. (**A**) The concentrations of IL-1β, IL-6, and TNF-α in the culture supernatant of HK-2 cells were detected by an enzyme-linked immunosorbent assay (ELISA) (*n* = 6). The cell was incubated with Sal (50 μM) and followed by stimulation with 1 ug/mL of LPS for 24 h. (**B**) Sal significantly suppresses the nuclear transport process of NF-κBp65 in LPS-induced HK-2 cells, as determined by immunofluorescence (*n* = 3). Original magnification: ×200. The cell was incubated with salidroside (50 μM) for 24 h. Then, LPS (1 μg/mL) stimulated the cell for 60 min. (**C**) Sal inhibited the LPS-induced increase in TLR4, p-IкBα, p-NF-κB, p-Ρ38, p-ERK, and p-JNK in the HK-2 cells, as determined by western blotting (*n* = 3). The cell was incubated with Sal (50 μM), followed by stimulation with 1 ug/mL of LPS for 24 h. All data are expressed as mean ± SD, ^#^ P < 0.01. ^##^ P < 0.01 vs. control group, * P < 0.05, ** P < 0.01 vs. LPS group.

In order to verify that salidroside works by inhibiting the TLR4/NF-κB and MAPK signaling pathways, we used lipopolysaccharide (LPS, a TLR4 receptor agonist) to stimulate the HK-2 cells. We detected the levels of inflammatory factors IL-1β, IL-6, and TNF-α in an LPS-induced HK-2 cell (Figure 7A). The results showed that inflammatory cytokines were notably increased in the model group and that Sal remarkably decreased the levels of IL-1β, TNF-α, and IL-6 in the culture supernatant of the HK-2 cells. In immunofluorescence, the effect of Sal (50 μM) on the nuclear transport process of NF-κBp65 in LPS-induced HK-2 cells was examined further (Figure 7B). As expected, Sal significantly suppressed the nuclear transport process of NF-κBp65 in LPS-induced HK-2 cells. The results of the western blot experiment showed that the expression of TLR4, p-IкBα, p-NF-κB, p-Ρ38, p-ERK, and p-JNK were significantly increased in the LPS group, while the salidroside (50 uM) treatment group effectively suppressed the expression of these proteins (Figure 7C). These results revealed that Sal suppresses TLR4-mediated inflammatory responses by inhibiting the TLR4/NF-κB and MAPK signaling pathways.

In summary, Sal could protect against RIF both in vivo and in vitro. These results confirm that Sal could inhibit the accumulation of the ECM and reduce the inflammatory response via the TLR4/NF-κB and MAPK pathways.

## 3. Discussion

**Figure 8:**
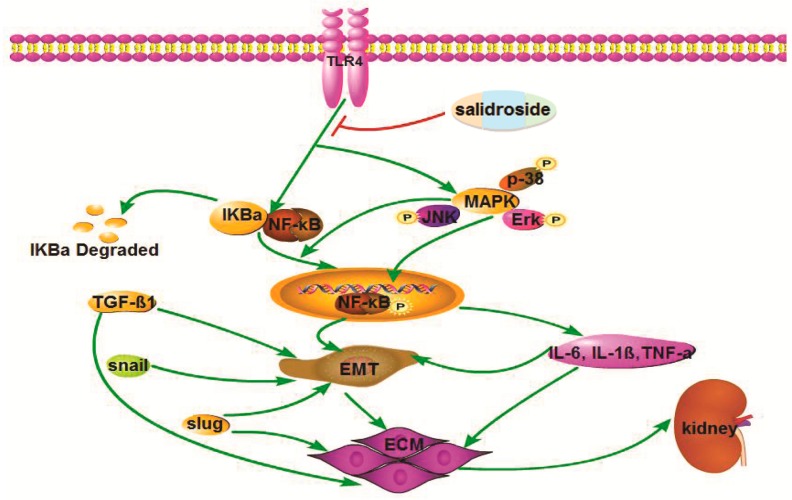
Schematic illustration of the role and mechanism of Sal when ameliorating renal fibrosis. Sal suppresses the TLR4/MAPK/NF-кB signaling pathways to inhibit the inflammatory response and EMT process.

Accumulating evidence suggests that Sal has many pharmacological activities. Specifically, Sal exhibits anti-oxidant and anti-inflammatory activities, both in vitro and in vivo. Our research reveals the anti-fibrosis effect of Sal and its potential mechanisms. The new findings are as follows: (1) Sal effectively inhibits the accumulation of the ECM and the EMT process in RIF. (2) Sal ameliorates renal fibrosis, both in vitro and in vivo. (3) Sal inhibits the inflammatory response and EMT process by suppressing the TLR4/NF-κB and MAPK signaling pathways, as illustrated in Figure 8.

Animal models of RIF provide an opportunity to explore underlying mechanisms and novel therapies. The UUO-induced RIF model has been widely used to mimic the pathological alterations of chronic obstructive nephropathy which are commonly observed in patients with CKD [22]. Complete ureteral obstruction is not a common cause of human renal disease. However, the UUO model is useful to examine the mechanisms of tubulointerstitial fibrosis in vivo [23]. This model can be induced in either rats or mice and shows no specific strain dependence. Complete UUO rapidly reduces renal blood flow and the glomerular filtration rate in the obstructed kidney within 24 h. The subsequent responses arise during the 7 days thereafter, which include interstitial inflammation (peak at 2 to 3 days), tubular dilation, tubular atrophy and fibrosis. The obstructed kidney reaches the end stage by around 2 weeks [23,24]. Folic acid induces RIF, and high dosages of folic acid (250 mg/kg) are given to mice to rapidly induce folic acid crystals, leading to tubular necrosis in the acute phase (1–14 days) and patchy interstitial fibrosis in the chronic phase (28–42 days). RIF is induced both by the crystal obstruction and the direct toxic effect to the tubular epithelial cells [25,26].

Most renal disorders result in renal fibrosis, so there is great interest in identifying the underlying factors behind this issue to prevent or reverse renal fibrosis. The proliferation of interstitial fibroblasts with myofibroblast transformation leads to excess deposition of the extracellular matrix and renal fibrosis [27]. The majority of CKDs are characterized by excessive ECM accumulation, including collagen I, collagen Ш and fibronectin [28]. In the tubulointerstitium of the kidneys, many cells are capable of producing the ECM, but fibroblasts are the principal matrix-producing cells that generate a large amount of interstitial matrix components. Fibroblasts in the kidneys are different, with SMA-positive myofibroblasts and express vimentin [29,30]. Snail is a key transcription factor that induces EMT, fibroblast migration and renal fibrosis. It is required for the development of fibrosis in renal epithelial cells. The reactivation of snail induces a partial EMT in tubular epithelial cells and promotes fibrogenesis, myofibroblast differentiation, and inflammation [26]. A large number of studies have shown that TGF-β1 is a key mediator and is highly upregulated in renal fibrosis in both experimental models and human kidneys [31]. TGF-β1 can promote extracellular matrix (ECM) production and inhibit its degradation to mediate progressive RIF. Furthermore, TGF-β1 has been identified to be the most potent inducer of EMT, which can induce tubular epithelial cells (TECs) to transform into myofibroblasts [32,33]. In our study, TGF-β1 was used to induce the transformation of HK-2 cells into myoblasts. Our results show that TGF-β1 can successfully induce EMT via activation of the TLR4/MAPK/NF-кB signaling pathways. Our results indicated that salidroside treatment significantly decreased the deposition of ECM and EMT markers, both in vivo and in vitro.

The correlation between fibrosis and inflammation has been established and supported by morphological evidence. A large number of studies have shown that the inflammatory response is necessary in the process of RIF [34]. Toll-like receptors (TLRs) are innate immune receptors that respond to endogenous danger factors and promote the activation of immune and inflammatory responses in RIF [35]. TLR4 is involved in systemic chronic diseases associated with inflammation, such as chronic kidney diseases, diabetes and metabolic syndrome [36]. TLR4, as the promoter of a signaling pathway, activates nuclear factor-κB (NF-κB) and stimulates pro-inflammatory responses. Some studies have revealed that the knockdown of the TLR4 significantly reduces the risk of fibrosis [37]. Renal fibrosis could be effectively ameliorated through inhibition of the NF-κB signaling pathway. In addition, findings have also indicated that direct contact between tubular and monocytes cells might be required to induce tubular EMT via a NF-κB-dependent pathway. Activating the NF-κB pathway could directly contribute to fibroblast activation and renal fibrosis [38]. Mitogen-activated protein kinases (MAPK) are intracellular signaling molecules that elicit diverse pro-inflammatory and profibrotic effects, both in vitro and in vivo. The anti-fibrosis effect has been reported recently in experimental models of RIF, showing that the blockade of the MAPK pathway ameliorated renal fibrosis. Several MAPK inhibitors have been developed and even applied in various stages of clinical trials. The JNK and Ρ38 pathways play important roles in the production of pro-inflammatory and profibrotic mediators, which are activated by various cellular stresses [39,40]. The administration of JNK and Ρ38 pharmacological inhibitors has been shown to suppress the development of glomerulosclerosis and tubulointerstitial fibrosis in various animal models [41]. Activated JNK and p38 also increase TGF-β1 gene transcription and induce enzyme expression that activates the latent form of TGF-β1 [42]. Many inflammatory cytokines (TNF-α, IL-1β, IL-6) can induce JNK, Ρ38, and NF-κB activation [40,43]. Tubular apoptosis is a typical feature of renal fibrosis [44]. The ERK pathway can ameliorate renal interstitial fibrosis through the suppression of tubular EMT [45]. The present study shows that Sal obviously suppresses the expression of TLR4, p-IкBα, p-NF-κB, p-Ρ38, p-ERK, and p-JNK, relieving the inflammatory response and ameliorating renal fibrosis.

In our study, we confirmed that Sal ameliorates renal interstitial fibrosis by inhibiting inflammatory cell infiltration and reducing the production of inflammatory cytokines. Moreover, Sal ameliorates renal fibrosis by reducing ECM accumulation and blocking the TLR4/NF-кB and MAPK signaling pathways. The results of this study provide new insights into reversing renal fibrosis through the anti-inflammatory effects of Sal. More studies are needed to further clarify the underlying mechanisms and enhance the performance of Sal in protecting RIF.

## 4. Materials and Methods

### 4.1. Main Reagents and Kits

Salidroside was provided by the Second Military Medical University (Shanghai, China; purity >99%). Folic acid and lipopolysaccharide (LPS) were purchased from Sigma Aldrich (St. Louis, USA). Recombinant human TGF-β1 was purchased from Pepro Tech (Rocky Hill, NJ, USA). Serum creatinine (Scr), blood urea nitrogen (BUN) and uric acid (UA) commercial kits were obtained from the Jiancheng Institute of Biotechnology (Nanjing, China). Enzyme-linked immunosorbent assay (ELISA) kits of interleukin (IL)-6, IL-1β and tumor necrosis factor (TNF)-α were purchased from Elabscience (Wuhan, China). The primary antibodies against α-SMA, vimentin, E-cadherin, snail, slug, NF-κB, p-IκBα, Erk, p-Erk, JNK, p-JNK, Ρ38, p-Ρ38, and GAPDH were produced by Cell Signaling Technology (Danvers, MA, USA). The anti-collagen I primary antibody was purchased from Affinity (Affinity Biosciences). The anti-collagen Ш primary antibody was obtained from Proteintech (Chicago, IL, USA). The anti-TLR4 and anti-TGF-β1 primary antibodies were produced by Santa Cruz Biotechnology (Santa Cruz, CA, USA). The anti-p-NF-κB and anti-IκBα primary antibodies were purchased from Abcam (Cambridge, UK). The antibodies are listed in Table S1 and the critical chemicals and commercial assays are listed in Table S2.

### 4.2. Animals and Experimental Design

All male C57BL/6 mice (age, 8–10 weeks; weight, 20–22 g) were purchased from the Jiangning Qinglongshan Animal Cultivation Farm (Nanjing, China) and were acclimated for 1 week before the experiment in the standard laboratory animal facility (25 °C, 12 h light/dark cycle) with free access to food and water. All studies were carried out in compliance with the Guide for the Care and Use of Laboratory Animals and the ethical guidelines of China Pharmaceutical University.

#### 4.2.1. UUO Model

Forty male mice were randomly divided into 4 groups (*n* = 10): (1) Sham group, (2) UUO group, (3) UUO + Sal (40 mg/kg) group, and the (4) UUO + Sal (80 mg/kg) group. The UUO or sham surgery was carried out under anesthesia with 3% chloral hydrate. Left proximal ureteral ligation was performed with 4-0 silk at two points and a cut was made between the points of ligation. The sham group had their ureters exposed and manipulated without ligation. Mice were given Sal at the corresponding dose by daily gastric gavage, beginning the first day after the surgical procedure. The sham and UUO groups were given identical volumes of saline. The mice were sacrificed on day 14 post-surgery for renal tissue and blood sampling. The blood was centrifuged (4000× *g*) for 10 min to obtain the serum, and the kidneys were harvested and then stored at −80 °C or fixed in a 4% paraformaldehyde solution.

#### 4.2.2. Folic Acid Model

Forty male mice were randomly divided into 4 groups (*n* = 10): (1) Control group, (2) FA group, (3) FA + Sal (40 mg/kg) group), and the (4) FA + Sal (80 mg/kg) group. The mice were injected with a single dose of folic acid (250 mg/kg, dissolved in 300 mM NaHCO3, i.p.), while the control group was injected with an identical voluminal vehicle (300 mM NaHCO3, i.p.). Mice were given Sal (40 and 80 mg/kg) at the corresponding dose by daily gastric gavage, beginning the first day after they were injected with FA. The kidney and blood samples were collected at 34 days post-FA injection.

### 4.3. H&E and Masson Staining

The mice were sacrificed by spinal dislocation. Kidney samples were excised immediately, fixed in 4% paraformaldehyde (PFA), then paraffin-embedded. The paraffin sections were dewaxed in xylene and dehydrated with ethanol, then stained with hematoxylin and eosin (H&E) to assess renal injury and Masson’s trichrome stains to assess collagen deposition. The observations were made under light microscopy (Nikon, Tokyo, Japan) at a 200× magnification.

### 4.4. Biochemical Assays in Serum

The levels of creatinine (Scr), uric acid (UA) and blood urea nitrogen (BUN) in the serum were measured by commercial kits according to the manufacturer’s instructions (Jiancheng Bioengineering Institute, Nanjing, China).

### 4.5. Inflammatory Cytokines Levels in Serum

The concentrations of IL-1β, IL-6, and TNF-α in the serum were determined by an enzyme-linked immunosorbent assay (ELISA) kit according to the manufacturer’s instructions (Elabscience, Wuhan, China).

### 4.6. Immunohistochemistry Staining

The expressions of collagen I, E-cadherin, vimentin and TLR4 in the kidney were evaluated by immunohistochemistry. Briefly, the kidneys were fixed with 4% PFA, embedded in paraffin and sliced into 5 µm thick sections. The sections were dewaxed and hydrated in graded ethanol, then microwaved in a sodium citrate buffer. The endogenous peroxidase activity was reduced using 3% H2O2 for 10 min. Each sample was blocked with 5% goat serum for 10 min and then treated with primary antibodies collagen I (1:100), E-cadherin (1:200), vimentin (1:200) and TLR4 (1:50) at 4 °C overnight. The next day, the sections were incubated with the goat anti-rabbit IgG secondary antibody for 10 min. Then, the sections were stained with 3,3-diaminobenzidine (DAB) and counterstained with hematoxylin. After dehydrating and drying, the sections were mounted with neutral gum and observed under a microscope.

### 4.7. Cell Culture

The HK-2 cells were obtained from the American Type Culture Collection and cultured in Dulbecco’s modified Eagle’s medium/F-12 nutrient mixture (DMEM/F12, NanJing KeyGen Biotech Co., Ltd., Nanjing, China), containing 10% fetal bovine serum (FBS, Gibco, Grand Island, NE, USA), 100 IU/mL penicillin and 100 IU/mL streptomycin in a humidified incubator containing 5% CO_2_ at 37 °C.

The HK-2 cells were seeded on 6-well-plate at a density of 2 × 10^5^ cell/well for 24 h. Afterwards, 5 ng/mL TGF-β1, with or without Sal (2, 10, 50 μM), stimulated the cells for 48 h. For further verification, LPS (1 μg/mL) and LPS plus Sal (50 μM) also stimulated the cells for 24 h. All the cells were then collected for the various analyses.

### 4.8. Western Blot

The kidney tissues and HK-2 cells were homogenated in an ice-cold RIPA buffer containing 2 mM PMSF. The samples were centrifuged at 12,000× *g* for 15 min at 4 °C and the total protein was determined by the BCA protein assay kit (Beyotime Biotechnology, Nanjing, China). The protein was separated by SDS-PAGE electrophoresis and then transferred to PVDF membranes. The membranes were blocked with 5% skim dried milk for 2 h and incubated with primary antibodies collagen I (1:1000), collagen Ш (1:1000), E-cadherin (1:1000), α-SMA (1:1000), vimentin (1:1000), TGF-β1 (1:200), snail (1:1000), slug (1:1000), TLR4 (1:200), IκBα (1:1000), p-IκBα (1:1000), p-NF-κB (1:1000), NF-κB (1:1000), Erk (1:1000), p-Erk (1:2000), JNK (1:1000), p-JNK (1:1000), Ρ38 (1:1000), p-Ρ38 (1:1000), and GAPDH (1:1000) at 4 °C overnight. After that, the membranes were incubated with the second antibodies (1:1000) for 2 h. The membranes were visualized using an ECL advanced kit and detected with a gel imaging system (Tanon Science & Technology Co., Ltd., Shanghai, China).

### 4.9. Cell Viability Assay

The HK-2 cells (1 × 10^4^ cells/well) were seeded in 96-well plates and incubated for 24 h, then treated with salidroside (0, 2, 10, 50 uM) for 24 h. Viability was assessed by the Cell Counting Kit-8 (CCK-8, Beyotime Biotechnology, Nanjing, China) assay. The data were evaluated as the percentage of the average absorbance to control group. Cell viability (%) = (A Treated / A Control) × 100%.

### 4.10. Immunofluorescence Staining

The expressions of NF-κBp65 in the HK-2 cells were evaluated by immunofluorescence. Briefly, the cell was washed three times with PBS, then fixed with an immunol staining fix solution (Beyotime Biotechnology, Nanjing, China) for 30 min and then blocked for 2 h with a blocking reagent. The cell was incubated with the primary antibody NF-κBp65 (1:200) overnight at 4 ℃, then washed three times with PBS and incubated with the goat anti-rabbit IgG (H+L) secondary antibody, the Alexa Fluor^®^ 488 conjugate (1:500), for 2 h. After washing three times with PBS and then with DAPI for 5 min, fluorescence images were captured with fluorescence microscopy.

### 4.11. Statistical Analysis

The experimental results are expressed as the mean ± standard deviation (SD). The statistical significance of differences between the means of each groups was analyzed by one-way ANOVA, followed by Tukey’s multiple comparison test. Differences in p-values less than 0.05 were considered statistically significant.

## Supplementary Materials

Supplementary materials can be found at https://www.mdpi.com/1422-0067/20/5/1103/s1.
