# Supplementary material for: Salidroside Ameliorates Renal Interstitial Fibrosis by Inhibiting the TLR4/NF-κB and MAPK Signaling Pathways
Source: Int J Mol Sci. 2019 Mar 4;20(5):1103. doi: 10.3390/ijms20051103 (PMC6429495; doi:10.3390/ijms20051103)
Supplement: Supplementary file 1 [file ijms-20-01103-s001.pdf]

Table S1. Antibodies.

Protein Name	Company	Identifier
Rabbit monoclonal anti-IκBα	Abcam	ab32518
Rabbit polyclonal anti-p-NF-κB p65	Abcam	ab86299
Mouse monoclonal anti-TLR4	Santa cruz	sc-293072
Rabbit monoclonal anti-TGF-β1	Santa cruz	sc-146
Rabbit monoclonal anti-p- IκBα	Cell Signaling Technology	#2859
Rabbit monoclonal anti-NF-κB p65	Cell Signaling Technology	#8242
Rabbit monoclonal anti-p-JNK	Cell Signaling Technology	#4668
Rabbit monoclonal anti -JNK	Cell Signaling Technology	#9258
Rabbit monoclonal anti-p-Erk	Cell Signaling Technology	#4370
Rabbit monoclonal anti-Erk	Cell Signaling Technology	#4695
Rabbit monoclonal anti-p-P38	Cell Signaling Technology	#4511
Rabbit monoclonal anti-P38	Cell Signaling Technology	#8690
Rabbit monoclonal anti -E-cadherin	Cell Signaling Technology	#3195
Rabbit monoclonal anti -Vimentin	Cell Signaling Technology	#5741
Rabbit monoclonal anti -slug	Cell Signaling Technology	#9585
Rabbit monoclonal anti -snail	Cell Signaling Technology	#3879
Rabbit monoclonal anti -α-SMA	Cell Signaling Technology	#19245
Rabbit monoclonal anti-GAPDH	Cell Signaling Technology	#2118
Rabbit polyclonal anti-collagen III	Proteintech	22734-1-AP
Rabbit polyclonal anti-collagen I	Affinity	AF7001
Anti-rabbit IgG, HRP-linked Antibody	Cell Signaling Technology	#7074
Anti-mouse IgG, HRP-linked Antibody	Cell Signaling Technology	#7076

Table S2. Critical Chemicals and Commercial Assays.

Reagents	Source	Identifier
Recombinant Human TGF-β1	Peprotech	100-21
Lipopolysaccharide	Sigma-Aldrich	L2880
Folic acid	Sigma-Aldrich	F7876
Dulbecco's modified Eagle medium/F12	NanJing KeyGen	KGM12500
Fetal bovine serum	Gibco	1600044
Trypsin-EDTA (0.25%)	Gibco	25200072
Serum creatinine assay Kit	Nanjing Jiancheng	C011-2
Blood urea nitrogen Assay Kit	Nanjing Jiancheng	C013-2
Uric acid assay kit	Nanjing Jiancheng	C012-2
Mouse TNF-α Elisa Kit	Elabscience	E-EL-M0049
Mouse IL-6 Elisa Kit	Elabscience	E-EL-M0044
Mouse IL-1β Elisa Kit	Elabscience	E-EL-M0037
Human TNF-α Elisa Kit	Elabscience	E-EL-H0109
Human IL-6 Elisa Kit	Elabscience	E-EL-H0102
Human IL-1β Elisa Kit	Elabscience	E-EL-H0149
